# Rotational coherence of encapsulated ortho and para water in fullerene-C_60_ revealed by time-domain terahertz spectroscopy

**DOI:** 10.1038/s41598-020-74972-3

**Published:** 2020-10-27

**Authors:** Sergey S. Zhukov, Vasileios Balos, Gabriela Hoffman, Shamim Alom, Mikhail Belyanchikov, Mehmet Nebioglu, Seulki Roh, Artem Pronin, George R. Bacanu, Pavel Abramov, Martin Wolf, Martin Dressel, Malcolm H. Levitt, Richard J. Whitby, Boris Gorshunov, Mohsen Sajadi

**Affiliations:** 1 Moscow Institute of Physics and Technology, Moscow, Russia; 2 Fritz-Haber-Institut der MPG, Berlin, Germany; 3 School of Chemistry, University of Southampton, Southampton, UK; 4 1. Physikalisches Institut, Universität Stuttgart, Stuttgart, Germany; 5 Department of Chemistry, University of Paderborn, Paderborn, Germany

**Keywords:** Physics, Atomic and molecular physics, Atomic and molecular interactions with photons

## Abstract

We resolve the real-time coherent rotational motion of isolated water molecules encapsulated in fullerene-C_60_ cages by time-domain terahertz (THz) spectroscopy. We employ single-cycle THz pulses to excite the low-frequency rotational motion of water and measure the subsequent coherent emission of electromagnetic waves by water molecules. At temperatures below ~ 100 K, C_60_ lattice vibrational damping is mitigated and the quantum dynamics of confined water are resolved with a markedly long rotational coherence, extended beyond 10 ps. The observed rotational transitions agree well with low-frequency rotational dynamics of single water molecules in the gas phase. However, some additional spectral features with their major contribution at ~2.26 THz are also observed which may indicate interaction between water rotation and the C_60_ lattice phonons. We also resolve the real-time change of the emission pattern of water after a sudden cooling to 4 K, signifying the conversion of ortho-water to para-water over the course of 10s hours. The observed long coherent rotational dynamics of isolated water molecules confined in C_60_ makes this system an attractive candidate for future quantum technology.

## Introduction

Resolving the real-time coherent dynamics of molecular systems is of central interest in physics and chemistry^1–3^. For example, the rotational quantum dynamics of isolated molecules have attracted considerable attention^4–12^. The relatively slow rotational motion of isolated molecules provides a detailed insight into the angular coordination of molecules and their decoherence trajectory, allowing optimization of the interaction potential energy surface of the molecular environment. Rotational motions with typically long coherence time are also considered as viable candidates for applications in quantum information processing^13,14^.

Among numerous molecular systems whose rotational quantum dynamics have been studied, the rotational dynamics of water are of particular interest because of its importance in atmospheric science and astrophysics, to name only two examples^15,16^. However, the rotational dynamics of water has a complex pattern because of (i) the low symmetry of water molecules -causing the irregular spacing of its rotational energy levels- and (ii) the constraint that is imposed on its rotational quantum wave function due to the symmetry of the wave functions of its spin isomers. The latter constraint is a consequence of quantum statistics^17^, according to which molecules with identical nuclei exist in the forms of spin isomers with distinct rotational quantum energy levels. As a result, water with two identical fermionic hydrogens forms two spin isomers, ortho-water with total nuclear spin of $I = 1$ and para-water with total nuclear spin of $I = 0$. Hence, the rotational dynamics of isolated water molecules are observed with contributions from both ortho- and para-water.

Studying the rotational dynamics of water enriched with only one form of its spin isomers is an ideal solution to reduce the complexity of the rotational pattern of water, but separation of ortho- and para- water is challenging. Despite some disputed claims^18^, the physical separation of the spin isomers of bulk water is not thought to be feasible^19,20^. Kravchuk et al.^21^ formed a magnetically focused molecular beam of ortho-water, based on the deflection of the nuclear magnetic moments and Horke et al.^22^ used strong static electric fields to gain spatial separation of the two isomers of water in a molecular beam. However, fast proton transfer in water limits the implementation of these salient approaches for resolving the real-time rotational dynamics of samples enriched with ortho- or para-water.

Kurotobi et al. made a breakthrough by encapsulating isolated water molecules in fullerene-C_60_ cages^23^. Since its birth, H_2_O@C_60_ has provided wealth of information on the quantum behavior of water, such as the conversion of ortho-water to para-water below 5 K, using various methods including NMR^24,25^, mid-IR^26^, FIR^23^, inelastic neutron scattering (INS)^23,27^ and dielectric measurements^28^. The INS also revealed the splitting of the triply-degenerate ortho-H_2_O rotational ground state into a singlet and a doublet state^23,24^.

The experimental data^22–25^ indicate that the lowest rotational states of water are almost unperturbed by the encapsulating cage at low temperature. The high symmetry of the cage allows water to rotate freely, with no perceptible hindering potential. This conclusion has been corroborated by nuclear magnetic resonance^29^, molecular dynamics (MD) simulations^30^ and multidimensional quantum calculations of the energy level structure of the encapsulated molecule^31,32^. Dielectric measurements of H_2_O@C_60_ indicate that the electric dipole moment of water is strongly screened by the cage, leading to a ~fourfold reduction in the effective dipole moment^25^, in good agreement with quantum chemistry calculations^32^. The most prominent influence of the molecular environment on the lowest rotational states of the encapsulated water is the breaking of the threefold degeneracy of the ortho-water ground state^23,24^, which has been attributed to an electric quadrupolar field generated by neighboring fullerene molecules in the lattice^33–35^.

While previous studies reveal the free rotation of encaged water molecules in C_60_ cages, the degree of rotational coherence of confined molecules can only be inferred by time-resolved spectroscopies^36^. Hence, our goal is to understand whether the encapsulated water in the highly symmetrical, homogenous and isolated environment of the inner space of C_60_ is able to rotate coherently, and if so, what is the coherence time of its rotation? Since there is no hydrogen binding between neighboring encaged water molecules, can the rotational coherence of water be resolved at cryogenic temperatures? Moreover, can we capture the conversion of the spin isomers of water through its real-time coherent rotational motions?

To obtain real-time rotational dynamics of molecules, non-resonant short laser pulses are often used to transiently align molecules, through which rotational wave packets are created. The signature of coherent rotational motions appears as periodically reviving features in the absence of the external field^37^. The revival signals are probed via measuring the induced optical birefringence in the ensemble. Alternatively, they can be resolved using strong ionizing laser pulses to break the molecular bonds and measure the angular distribution of the corresponding fragments^38^. However, neither laser induced alignment, nor the two probing techniques would be the proper choices to study the alignment dynamics of encapsulated molecules inside C_60_ cages. The high optical absorption coefficient of solid C_60_ ($\alpha \approx 10^{4} \; {\text{cm}}^{ - 1}$ @ 650 nm^39^) makes the optical-induced alignment of the encapsulated molecules very challenging. Moreover, even if the encapsulated molecules are selectively fragmented, ions may not escape their cages.

Fortunately, fullerene-C_60_ is transparent at THz frequencies and short THz pulses can be generated to resonantly create rotational wave packets in water molecules. Hence, we employ time-domain terahertz spectroscopy to resolve the rotational dynamics of encapsulated water inside C_60_ with sub-picosecond temporal resolution. In the current work, we use only weak THz pulses to resolve the coherent orientational motion of water molecules; in a forthcoming publication, we address the nonlinear THz response of H_2_O@C_60_.

## Experimental details

### H_2_O@C_60_ Sample

80% filled H_2_O@C_60_ was synthesized as described earlier^40^, intimately mixed with 15 times its mass of commercial 99.5% C_60_ (MTR Ltd) and the mixture sublimed in a quartz tube at 10^−5^ to 10^−6^ torr at 550 °C (with a 10 °C/min temperature ramp). The product was assayed for composition by HPLC of a sample on a Cosmosil BuckyPrep analytical column (4.6 × 250 mm) using toluene as the eluent at 1 ml/min which confirmed the desired 5% H_2_O filling. Our sample characterization (detailed in Ref. 40) clearly shows the isolation of single H_2_O molecules inside C_60_ cages.

### Time-domain THz spectroscopy

**Figure 1:**
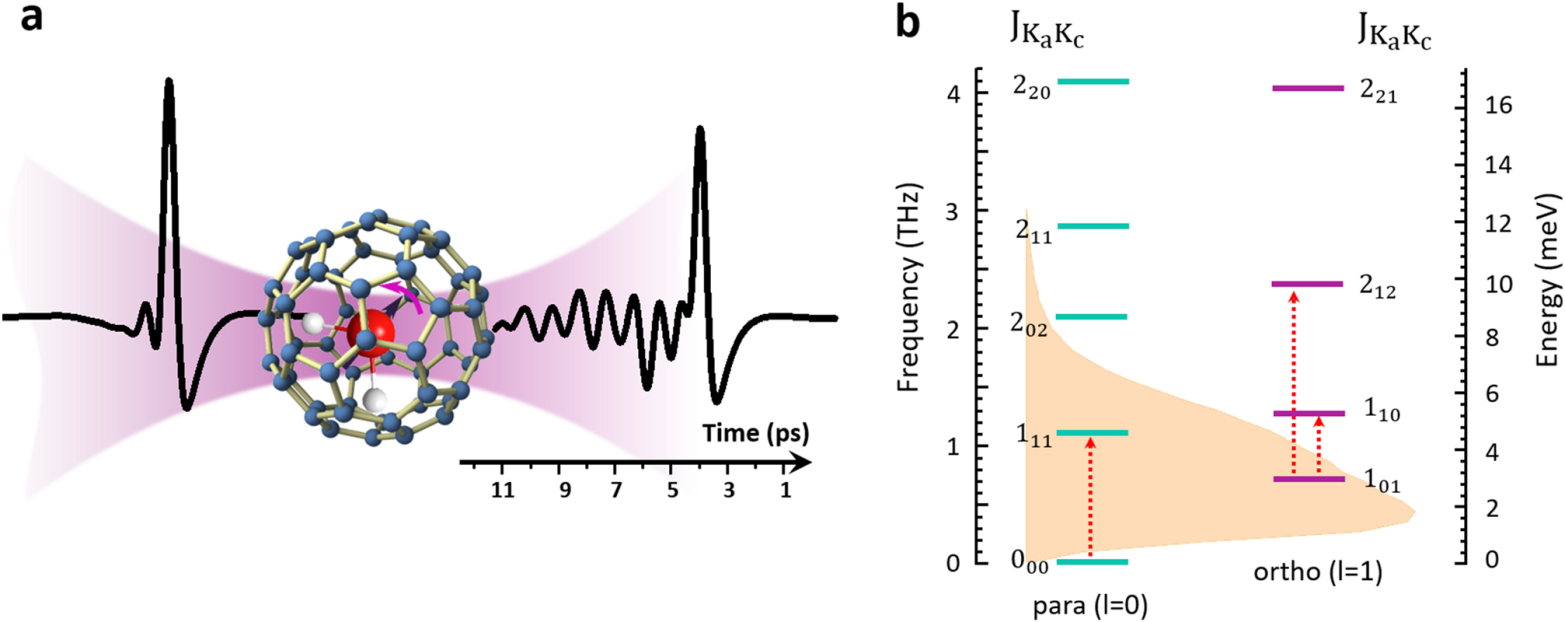
THz time-domain spectroscopy of H_2_O@C_60_. a, A single cycle-THz pulse traverses the H_2_O@C_60_ sample and launches a rotational wave packet in the rotational quantum states of encapsulated water molecules. The coherent radiation of the molecules is observed as the tail of the transmitted THz pulse. b, The lowest rotational energy levels of water molecules. The para and ortho energy levels are labelled separately by the quantum numbers ${\text{J}}_{{{\text{K}}_{{\text{a}}} {\text{K}}_{{\text{c}}} }}$ (see text for details). The amber-colored area shows the frequency spectrum of the THz pump pulse. The arrows indicate the three lowest rotational transitions of water.

A schematic of the time-domain THz spectroscopy experiment is illustrated in Fig. 1a. A THz pulse generated via photoconductive switching technique, with center frequency of ~ 0.5 THz, temporal duration of 0.5 ps and the bandwidth of ~ 1.5 THz is incident with the H_2_O@C_60_ sample. The amber colored area in Fig. 1b shows the amplitude spectrum of the pulse. The transmitted THz pulse and the coherent emissions of water molecules are measured using short laser pulses in a reverse photo-conductive switching process with temporal resolution of ~ 2 fs. The H_2_O@C_60_ sample is a bi-layer pellet, whose first layer (~0.2 mm thick) is a homogeneous mixture comprising 5% of H_2_O@C_60_ and 95% empty C_60_ and the second layer is empty C_60_ (~0.5 mm thick). The diluted 5% H_2_O@C_60_ is used to avoid saturation of the absorption transitions and the large thickness of the bi-layer pellet helps to delay the first reflected echo signal from the pellet-air interface to ~ 8 ps; opening a wide temporal window for capturing a large fraction of the water’s coherent emissions, without their interference with the reflected echo signal. Note that although very thin 100% H_2_O@C_60_ samples with ~10 $\upmu {\text{m}}$ thickness can also be used to obtain the unsaturated rotational transitions, freestanding thin films of C_60_ are very fragile and challenging to work with.

We use two commercial THz spectrometers namely, TeraPulse and TeraView to perform the experiments. The sample is cooled down to liquid helium temperatures in an Optistat (Oxford Instruments) helium-flow optical cryostat with Mylar windows. The THz pulse path is purged by nitrogen or vacuumed to exclude water vapor absorption lines. Each signal is obtained by averaging of about 200–500 pulses and accumulating for 30–40 seconds. The step size of the time-delay line movement provides a temporal resolution of 3 fs on the TeraView spectrometer and 8 fs on the TeraPulse spectrometer. In the temperature dependent measurements, the sample temperature is gradually lowered and the THz response of the system is measured at 10-degree steps and after 60 min waiting time.

## Results and discussion

Upon propagation of a weak THz pulse with electric field ${\mathbf{E}}\left( t \right)$ through the H_2_O@C_60_ sample, a linear polarization ${\mathbf{P}}\left( t \right) = \chi \left( t \right){\mathbf{E}}\left( t \right)$ is formed. Here, $\chi \left( t \right)$ is the electric susceptibility of the sample and describes the temporal build-up and decay of ${\mathbf{P}}\left( t \right)$. The induced polarization is related to the orientation of molecules upon THz excitation via $P\left( t \right) = N \mu {\cos}\theta \left( t \right)$, where $N$ is the number density, $\mu$ is the permanent dipole moment of water molecule and $\theta$ is the angle between the THz field polarization axis and $\mu$. As the THz pulse resonantly excites the rotational motion of water, ${\mathbf{E}}\left( t \right)$ directly couples into the molecular dipole moment and exerts a ${{\varvec{\upmu}}} \times {\mathbf{E}}\left( t \right)$ torque on water molecules, hence time-domain THz spectroscopy gains high sensitivity to explore details of the rotational motion of encapsulated water inside its THz transparent C_60_ cage. The potential energy of this light-matter interaction is explained by the Hamiltonian $H_{1} = - {{\varvec{\upmu}}}\cdot{\mathbf{E}}\left( t \right) = - \mu E\left( t \right) {\cos}\theta$. This interaction induces a coherent wave-packet in the rotational quantum states of water, such that one-quantum coherences (1QCs) are induced between the rotational eigenstates. The induced 1QCs cause the emission of electromagnetic waves whose amplitude decays exponentially, known as a free-induction-decay (FID)^11,41^.

As an asymmetric top molecule, water has three distinct moments of inertia $I_{a}$, $I_{b}$ and $I_{c}$ along the three principal axes of its inertial tensor and its Hamiltonian is given by $\hat{H}_{0} = \frac{{\hat{J}_{a}^{2} }}{{2I_{a} }} + \frac{{\hat{J}_{b}^{2} }}{{2I_{b} }} + \frac{{\hat{J}_{c}^{2} }}{{2I_{c} }}$, where $\hat{J}_{i}$ is a component of angular momentum operator along $i = a,b,c$. As the molecules are initially distributed in different $J$ states, the phase relation induced by the THz pulse is quickly lost and their FID emissions diphase and interfere destructively. After a period known as revival period ${\text{T}}_{{{\text{rev}}}} = 1/2B_{i} c$ ($c$ is the speed of light and $B_{i} = \hbar^{2} /2I_{i}$ is the rotational constant corresponding to each principle axis) the dipoles reorient and their FID emissions interfere constructively. Note that, as water has three rotational constants, the molecular rotational motion gains a complex pattern. To label the rotational transitions of the asymmetric top molecules, we follow the procedure in which, each rotational energy level is denoted by the $J$ state and its projections, ${\text{K}}_{{\text{a}}}$ and ${\text{K}}_{{\text{c}}}$ along the principal axes $a$ and $c$ ^42^.

According to the Pauli principle, for water with identical fermionic protons, the total internal wave function is antisymmetric with respect to particle exchange^43^. For such system in its ground electronic and vibronic state, the wave function $\left| \psi \right. \rangle = \left| {\psi_{{{\text{rot}}}} } \right. \rangle \left| {\psi_{{{\text{spin}}}} } \right. \rangle$ changes sign by the permutation of its hydrogen atoms. As a result, for para-water with antisymmetric nuclear-spin wave function $\left| {\psi_{{{\text{spin}}}} } \right. \rangle$, the only allowed spatial wave functions are symmetric with respect to particle exchange. These are characterized by even values of K_a_ + K_c_. In contrast, for ortho-water with symmetric $\left| {\psi_{{{\text{spin}}}} } \right. \rangle$, the allowed spatial wave functions are antisymmetric with respect to particle exchange, corresponding to odd values of K_a_ + K_c_. As depicted in Fig. 1b, the absolute rotational ground state of water belongs to para-water ${\text{J}}_{{{\text{K}}_{{\text{a}}} {\text{K}}_{{\text{c}}} }} = 0_{00}$ with $1_{11}$ and $1_{02}$ as its first and second excited rotational states. The ground rotational state of the ortho-water is ${\text{J}}_{{{\text{K}}_{{\text{a}}} {\text{K}}_{{\text{c}}} }} = 1_{01}$ and $1_{10}$ and $1_{12}$ are the first two excited rotational states.

### Impact of temperature

**Figure 2:**
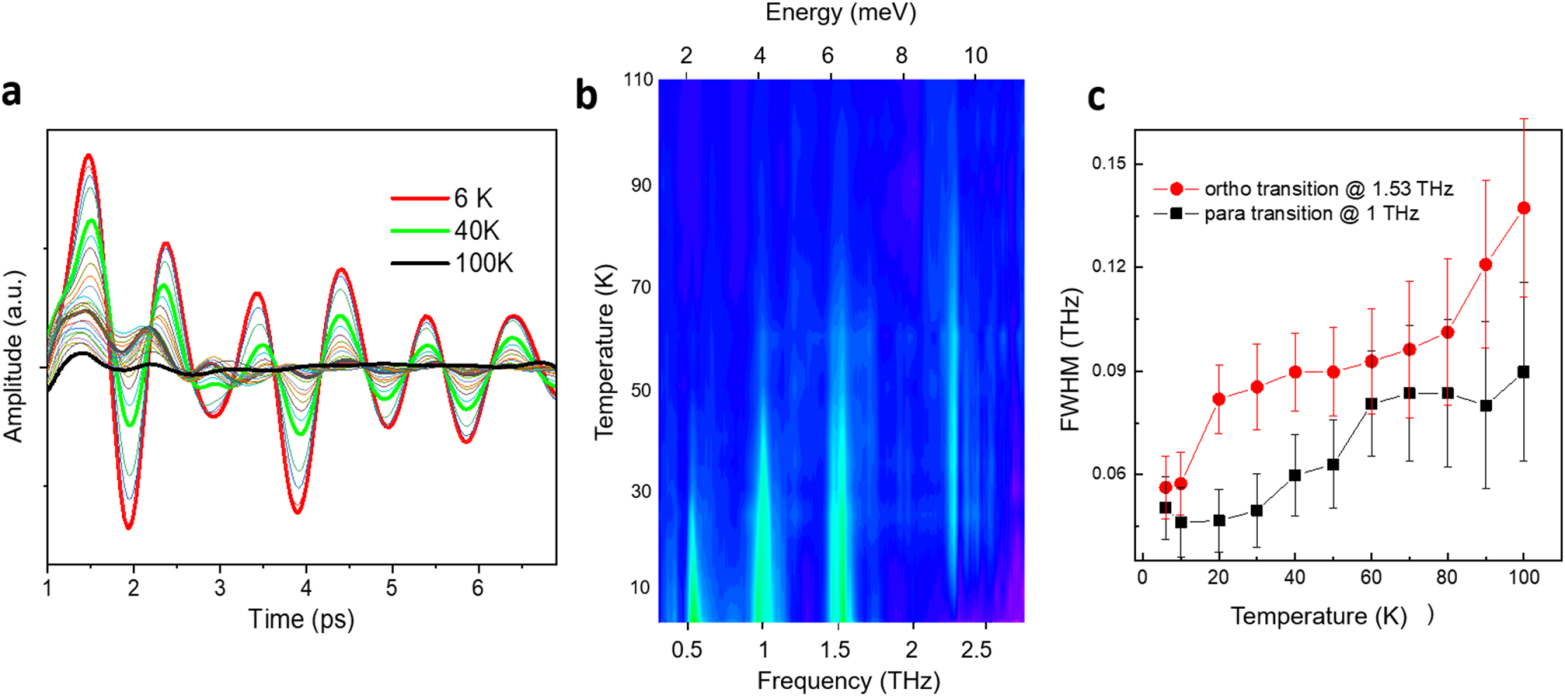
Temperature-dependent rotational motion of H_2_O@C_60_. a, The rotational coherences of H_2_O are clearly resolved at cryogenic temperatures, but display increasing damping as the temperature is increased. b, The contour plot of the Fourier spectra of the time-domain signals shows the rotational components (the bright lines) of the encapsulated water molecules as function of sample temperature. c, The rotational transitions at 1 THz and at 1.53 THz clearly show an increase in linewidth with increasing temperature.

First, we study the impact of temperature, in the range of 300 K to 6 K, on the THz response of the encapsulated water. The H_2_O@C_60_ response, subtracted from that of the empty C_60_ and their corresponding Fourier spectra are presented in Fig. 2a,b (see raw data before subtraction in Fig. S1). As discussed below, the observed oscillations are the FID emissions of water inside C_60_. While at higher temperatures no emission is detected, the onset of FID emissions is resolved at temperatures below ~100 K. The amplitude of the signals increases on further cooling of the sample.

As shown in Fig. 2b, the emission encompasses different spectral components, with first three low-frequency contributions centered at ~0.52 THz, ~1 THz and ~1.53 THz, corresponding to the transitions with quantum energies 2.15 meV, 4.14 meV and 6.33 meV, respectively. The observed rotational transitions are in good agreement with the three lowest rotational transitions of free water^44^: the para transition $0_{00} \to 1_{11}$ with energy 4.56 meV, and two ortho transitions $1_{01} \to 1_{10}$ and $1_{01} \to 2_{12}$ with energies 2.26 meV and 6.85 meV (the three transitions are marked by red dashed arrows in Fig. 1b).

The difference between the rotational frequencies of the free water and those of the encapsulated water, from the lowest to highest frequencies, are -1.2 cm^−1^, + 4 cm^−1^ and + 4.6 cm^−1^, implying only a minor impact of the C_60_ cage on the energy levels of the encapsulated water molecules, consistent with previous FIR and INS findings^23^. In addition to the major three low-frequency rotational transitions, there are bright lines in the contour plot of Fig. 2b, at frequencies higher than 1.5 THz, with the brightest one at 2.26 THz.

**Figure 3:**
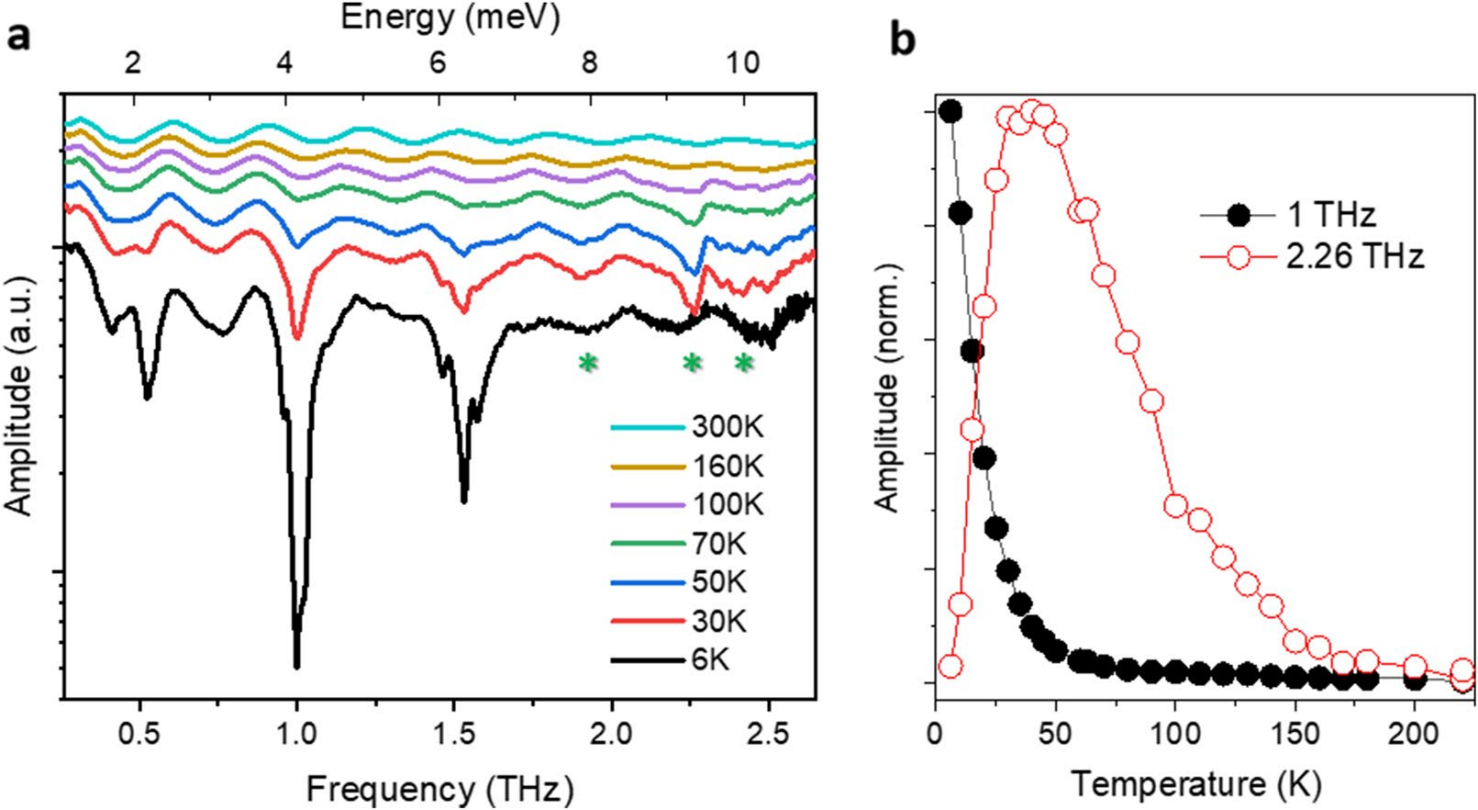
Temperature-dependent rotational spectra. a, Selected Fourier spectra of the time-domain FID emissions of H_2_O@C_60_. The prominent peaks at 0.52 THz, 1 THz and 1.53 THz monotonically gain larger amplitude by lowering temperature and match well with rotational transitions of free water. The peak at 2.26 THz and two broad features at about 1.9 THz and 2.49 THz, marked by green asterisks, do not match the free water transitions (see text for details). The spectra are vertically displaced for clarity. Note the logarithmic scale of the vertical axis. Periodic oscillations are due to the Fabry–Pérot effect. b, The peak amplitude of the transitions at 1 THz and 2.26 THz, normalized against their maximum values.

The selected spectra of the contour plot are displayed in Fig. 3a and clearly show a relatively strong peak at 2.26 THz and two weak broad features, marked by asterisks, at about 1.9 THz and 2.49 THz. Interestingly, as depicted in Fig. 3b, while the three low-frequency peaks (presented by the one at ~ 1THz) monotonically gain larger amplitude by lowering temperature, the peak at 2.26 THz gains its largest amplitude at about 50 K and completely vanishes below ~10 K.

To understand the temperature behavior of coherent FID emissions of water inside C_60_, we digress here to consider the C_60_ lattice structure and dynamics as function of temperature. Pure C_60_ adopts face-centered cubic crystal structure at room temperature. At about 255 K, it undergoes a first-order phase transition, accompanied by a small contraction of lattice constant (14.1501 Å $\to$ 14.1015 Å) and forms simple cubic structure^45,46^. By further cooling a second-order phase transition occurs at 90 K^47^.

The dynamics of C_60_ lattice have both intra- and inter-molecular contributions. With a relatively large band gap, the intramolecular dynamics of C_60_ lattice cover the energy range above ~30 meV, whereas the low-frequency intermolecular phonons have maximum energy of ~ 6.4 meV^48^. The latter region is dominated by translational and rotational (librational) motions. At room temperature, C_60_ molecules perform continuous rotational diffusion. In the range 267 – 90 K molecules perform random jump between symmetry-equivalent orientations. In the very cold regime below 90 K phonons become less active^47^.

The absence of FID emissions at temperatures above ~100 K can straightforwardly be attributed to the strong intermolecular C_60_ lattice dynamics, such that the coherent rotation of water is completely damped by frequent collisions of the rotating encapsulated water molecule with its cage. Notably, the absence of such strong lattice-damping effect in water vapor results in long coherent rotational time of ~100 ps^49,50^. By lowering the temperature, the encaged water molecules experience less fluctuations, hence the THz-induced rotational coherence of water lasts for a longer time. As a result, the FID emissions of water molecules can be resolved below T $\approx$ 100 K.

The damping effect of the environment on water rotational dynamics can also be realized from the broadening of the rotational transitions. As shown in Fig. 2c, the full width at half maximum (FWHM) of the two strong bands at 1 THz and 1.53 THz increase as the temperature is increased from 6 K to about 100 K. The rapid increase in linewidth for the ortho-H_2_O transition above ~ 80 K might be associated with a phase transition in C_60_, with increased rotational freedom of the C_60_ cages themselves above ~90 K^47^.

The fact that the transition at 2.26 THz only appears at higher temperatures strongly suggests that it originates from an excited rotational state. The selection rules for electromagnetic transitions are as follows: $\Delta {\text{J}} = 0, \pm 1$, $\Delta {\text{K}}_{{\text{a}}} = \pm 1$, $\Delta {\text{K}}_{{\text{c}}} = \pm 1$. Accordingly, the allowed transitions, originating from the first rotational excited states, are (the frequencies in isolated water molecules, from Ref.^49^ are given in brackets): *para* $1_{11} \to 2_{02}$ (0.99 THz), *para* $1_{11} \to 2_{11}$ (1.74 THz), *para* $1_{11} \to 2_{20}$ (2.97 THz) and *ortho* $1_{10} \to 2_{21}$ (2.77 THz). However, none of these frequencies matches closely the observed 2.26 THz transition. The closest candidate is the ortho $1_{10} \to 2_{21}$ transition, but even this would require a red shift of ~ 15 cm^−1^ with respect to free water. A red shift of this magnitude is far larger than those predicted by quantum calculations^27^ and in contrast to the negligible difference between the three lowest energy levels of the free water and the encapsulated water. In the same way, the broad features at ~1.9 THz and ~2.49 THz would require, respectively a blue shift by ~30 cm^−1^ and a red shift by ~15 cm^−1^ to match the closest free water transition of $1_{11} \to 2_{02}$ and $1_{11} \to 2_{20}$.

At this point, the origin of the 2.26 THz peak is unknown. One possibility is that the potential energy function used for the existing quantum calculations^27,31,32^ is insufficiently accurate to predict the behavior of the excited rotational states of the encapsulated water. Another possibility is that the 2.26 THz peak involves an interaction between the water rotation and the lattice phonons. Neutron scattering studies have indicated the presence of low-frequency phonons in the C_60_ lattice at about 2 THz^51^. Hence, the low-frequency phonons of the C_60_ lattice, which are normally invisible to the THz radiation, might become visible due to their coupling to the water dipole.

It is also very instructive to compare the relaxation of electric polarization ${\mathbf{P}}\left( t \right)$ of H_2_O@C_60_ with that obtained from molecular dynamics simulations. We determine the relaxation time constants of ${\mathbf{P}}\left( t \right)$ as function of temperature by fitting the measured time domain signals in Fig. 2a using the decaying oscillatory functions $\mathop \sum \limits_{i} A_{i} e^{ - \gamma t} {\sin}\left( {2\pi \nu_{i} t} \right)$. As shown in Fig. S2, the decay time constants of the polarization $T_{2} = \gamma^{ - 1}$ increase from ~2 ps at about 90 K to ~3.8 ps at 6 K. In contrast, the results of MD simulations performed by Ensing et al.^32^, using ab initio density functional theory (DFT) shows much faster relaxation. The authors computed the orientational correlation functions $P_{l} \left( {\cos \theta } \right)$ at a range of temperatures, where $P_{l} \left( x \right)$ is the rank-$l$ Legendre polynomial and the angle $\theta$ describes the orientation of the water dipole moment in H_2_O@C_60_. The correlation function for $P_{2} \left( {\cos \theta } \right)$ was estimated to decay approximately exponentially with a time constant of ~ 100 fs at 50 K and 50 fs at 100 K. Assuming a rotational diffusion model of the water rotation, the rank-1 correlation function $P_{1} \left( {\cos \theta } \right) = \cos \theta$ is expected to decay with a time constant 3 times longer, i.e. ~300 fs at 50 K and 150 fs at 100 K. The salient point is that these time constants, predicted by molecular dynamics using a force field estimated by DFT, are much shorter than those observed experimentally by time-domain THz spectroscopy. As shown also in Fig. S3, the correlation decay time constant of 150 fs at 100 K estimated by molecular dynamics is more than one order of magnitude shorter than the decay time constant of the rotational coherences, observed experimentally. This large discrepancy between the MD calculations and the experimental THz results may be attributed to a low-temperature quantum nuclear effects in the rotational dynamics, which is beyond the scope of the MD calculations. As remarked by the authors of Ref.^32^, “at low temperatures quantum rotation of the water molecule becomes significant, which is not captured by our method”. The results shown in Fig. 2a, and in particular the long decay of the rotational coherences, therefore may reveal the classical-to-quantum transition for the rotation of the encapsulated water molecules.

The latter dynamical contrast may be attributed to the different energy exchange mechanism of quantum- vs classical-rotors with their environment. For a classical-rotor the energy between water and its environment is exchanged upon each collision of water and the cage inner surface, whereas for a quantum-rotor the energy exchange follows matching of the energy levels of the rotor and that of the C_60_ lattice.

### Ortho-to-para conversion

**Figure 4:**
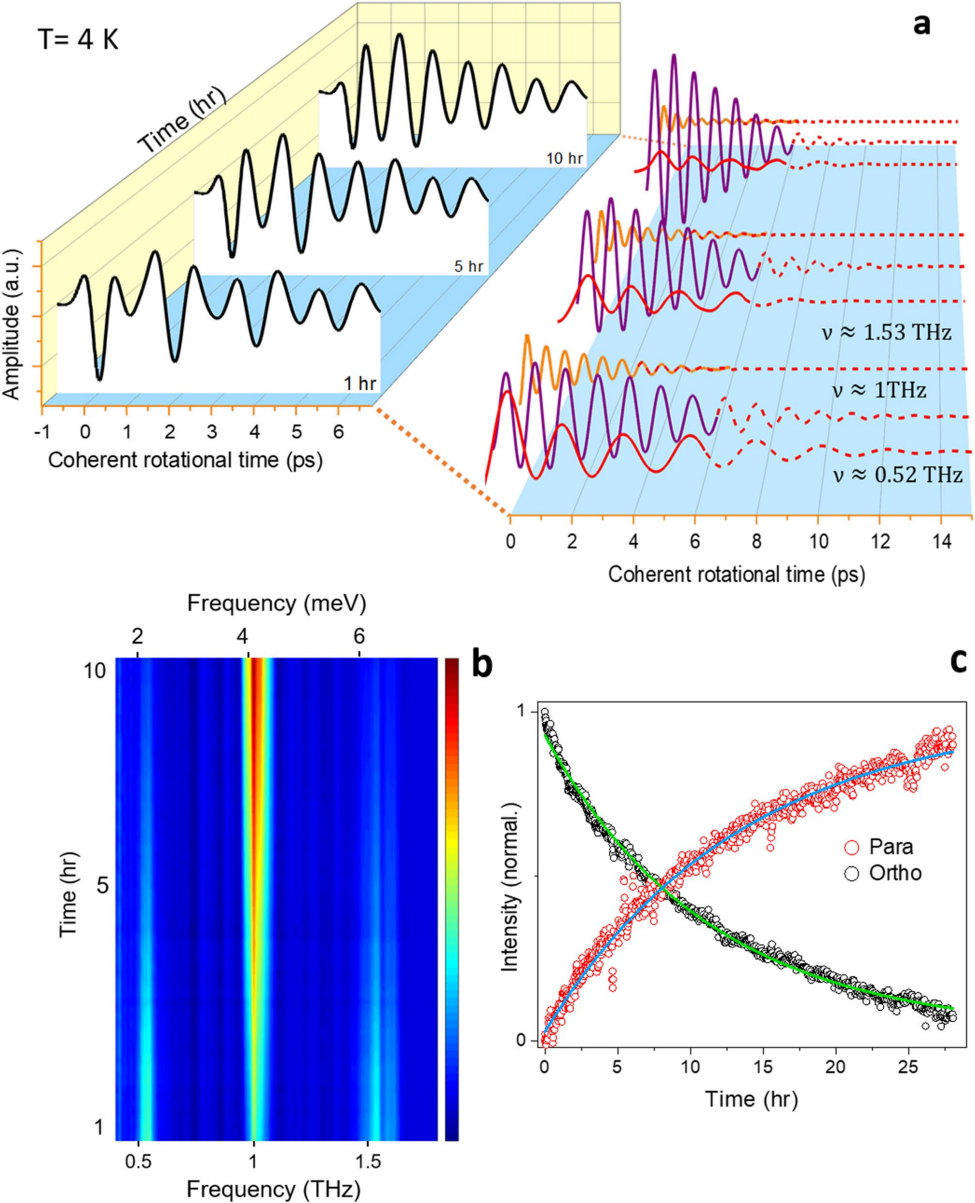
Ortho-para spin conversion of water at 4 K. (**a**) The rotational motion of the encapsulated water in C_60_ is measured as function of waiting time after a sudden cooling of H_2_O@C_60_ from 20 to 4 K. The non-equilibrium population ratio of ortho and para spin isomers is directly observed by enhancing one oscillation at 1 THz and the reduction of frequency components at 1.53 THz and 0.52 THz. In the left panel, one observes the change of the pattern of the FIDs in the course of 10 h. In the right panel, each trace is decomposed into its three frequency components. The dashed lines are extrapolations beyond 8 ps. (**b**), The Fourier spectra of the time-domain rotational signal in panel a. In the time window of 10 h the ortho lines at ~0.52 THz and ~1.53 THz lose their amplitude (coded dark blue), while the para line at ~1 THz gains larger amplitude (coded red). (**c**) The kinetics of the amplitude of the para-water (@ 1THz) and ortho-water (@ 0.52 THz). The solid lines are single exponential fits to the experimental data (see text for details).

Second, we measure the FID emissions of the encapsulated water at 4 K, over the course of ~ 30 h. For these experiments, we gradually lowered the sample temperature to 20 K, and then rapidly lowered it to 4 K. The FID signals at waiting times equal to 1, 5 and 10 h are shown in the left panel of Fig. 4a. An interesting observation is the gradual change of the FID emission pattern, from a more complex to a simpler pattern. We determine the FID emission components by fitting the measured signals with $\mathop \sum \limits_{i} A_{i} e^{ - \gamma t} {\sin}\left( {2\pi \nu_{i} t} \right)$, where $\nu_{i}$ and $\gamma$ are respectively, the frequency and the damping constant of the fitted components^52^. The signals can be described reasonably well by three decaying oscillations with $\nu_{1} \approx 0.52\;{\text{THz}}$, $\nu_{2} \approx 1\;{\text{THz}}$, $\nu_{3} \approx 1.53\;{\text{THz}}$ and $\gamma \approx 0.26\;{\text{ps}}^{ - 1}$ (see Fig. S3). The fitted components are shown in the right panel of Fig. 4a. The dashed lines are extrapolations of the fted curves beyond 8 ps. The change of the FID components over the course of 10 h can be clearly seen. In this temporal window, the amplitude of the components $\nu_{1} \approx 0.52\;{\text{THz}}$ and $\nu_{3} \approx 1.53\;{\text{THz}}$ is reduced, whereas in contrast, the component $\nu_{2} \approx 1\;{\text{THz}}$ gains larger amplitude. The amplitude variations can also be seen in the Fourier spectra of the FID signals of the H_2_O@C_60_ in Fig. 4b, where the two ortho transitions lose their strength, while the para component becomes brighter. The temporal evolution of the latter amplitude variation, taken from the ortho component $\nu_{1}$. and the para component $\nu_{2}$, is given in Fig. 4c. Both curves can be fit well by single exponentials with ortho decay time of 11.22 $\pm$ 0.14 h and para rise time of 13.5 $\pm$ 0.25 h, in general agreement with previous findings^27,30^. We attribute the difference between the rise and decay time constants to the termination of the measurement prior to reaching the equilibrium state.

These results manifest the real-time conversion of ortho-to-para spin isomers of water. They demonstrate that the coherent rotation of entrapped water molecules lasts for about 15 ps inside its cage at 4 K, to the best of our knowledge the longest coherent rotational motion observed for confined molecules in nano-cages. The damping of the rotational coherence may be attributed to the shaking of the C_60_ cage due to lattice vibrations which couple to the rotational motion of water molecules through translation-rotation coupling^26,31^. Since we used a diluted 5% H_2_O@C_60_ sample, water-water interactions may be ruled out as the damping mechanism of the rotational coherence.

## Conclusion and outlook

In conclusion, using single cycle THz pulses we launched coherent wave packets in the low-frequency rotational transitions of an ensemble of H_2_O@C_60_ (encapsulated water in fullerene-C_60_) and resolved the coherent emission of electromagnetic waves by the oriented dipoles. Underdamped coherent emissions are observed at T < 100 K, the temperature below which the lattice vibrations are mitigated. At 4 K, the lowest temperature we reach, the rotation of water attains a long coherent trajectory extended beyond 10 ps. This is far longer than the rotational correlation time predicted by molecular dynamics simulations, indicating a transition from classical to quantum rotation of the water molecules at low temperature. Although this coherence decay time for the molecular rotation is unusually long for encaged molecules, this decay time is still shorter than that observed for water in the gaseous state at room temperature. We also directly observe the change of the pattern of the dipole emission at 4 K, from a mixture of ortho- and para-water to a more purified para emission after a waiting period of 10s of hours, indicating the inter-conversion of spin isomers of water at cryogenic temperatures.

It would also be of interest to probe the quantum state lifetimes of the H_2_O@C_60_ rotational transitions. In the case of optically transparent samples, these lifetimes may be probed by THz pump / optical probe measurements^11,53–55^. However, this is not possible for the current case, since the black powders of H_2_O@C_60_ are optically dense. An alternative method is to use THz pump / THz probe experiments, which we will address in a forthcoming publication.

The coherent molecular rotational motions are relevant to a wide variety of research disciplines. Dynamics of water in close vicinity of hydrophobic surfaces and inside nano-cavities are important for heterogeneous catalysis and studies related to the behavior of confined water in biological systems. The relaxation rate of water’s rotational coherence inside C_60_, free from hydrogen binding may also be used as an ideal model system to obtain an accurate estimate for the interaction potential of water with a hydrophobic surface.

The coherent rotational dynamics of water in C_60_ may find future application in quantum information processing. The solid nature of H_2_O@C_60_ and the tunability of the frequency, the phase and the polarization of THz pulses^53^ facilitate the miniaturization and integration of rotational coherence of water within electrical circuits^56,57^, and pave the way for coherent control and selective excitation of molecular rotational motions. Although the rotational coherence of water inside C_60_ is too short for quantum computing purposes, unique features gained by the combination of THz pulses and the solid structure of H_2_O@C_60_ may encourage further research to find longer coherent dynamics of encaged molecules. These studies may shed further light on the damping mechanism of the rotational coherences of encapsulated water molecules by their cages, for example, by replacing the empty C_60_ molecules by another host media such as CS_2_ or by disturbing the C_60_ lattice structure by introducing co-crystalline compounds such as benzene or porphyrin^58,59^.

### Associated content

#### Supporting Information

THz response of H_2_O@C_60_ before subtraction from the empty C_60_ response, polarization relaxation of water encaged inside C_60_ as function of temperature, and the comparison between the polarization relaxations obtained from the classical MD simulations and those obtained from the fit to the experimental FID emissions.

## Supplementary information

Supplementary Information
