# Supplementary material for: Rotational coherence of encapsulated ortho and para water in fullerene-C60 revealed by time-domain terahertz spectroscopy
Source: Sci Rep. 2020 Oct 27;10:18329. doi: 10.1038/s41598-020-74972-3 (PMC7592058; doi:10.1038/s41598-020-74972-3)
Supplement: Supplementary file 1 — Supplementary Information [file 41598_2020_74972_MOESM1_ESM.pdf]

Supporting Information

Rotational Coherence of Encapsulated Ortho and Para Water in Fullerene-C_{60} Revealed by Time-Domain Terahertz Spectroscopy

Sergey S. Zhukov1[†], Vasileios Balos[2†], Gabriela Hoffman[3], Shamim Alom[3], Mikhail Belyanchikov[1], Mehmet Nebioglu[4], Seulki Roh[4], Artem Pronin[4], George R. Bacanu[3], Pavel Abramov[1], Martin Wolf[2], Martin Dressel[1,4], Malcolm H. Levitt[3], Richard J. Whitby[3], Boris Gorshunov[1*], Mohsen Sajadi[2,5*]

[1]*Moscow Institute of Physics and Technology, Moscow, Russia.*

[2]*Fritz-Haber-Institut der MPG, Berlin, Germany.*

[3]*School of Chemistry, University of Southampton, Southampton, United Kingdom.*

[4]*1.Physikalisches Institut, Universität Stuttgart, Stuttgart, Germany.*

[5]*Department of Chemistry, University of Paderborn, Paderborn, Germany.*

[†]These authors contributed equally in this work.

Email: bpgorshunov@gmail.com & *sajadi@fhi-berlin.mpg.de.*

THz response of $H_2O@C_{60}$ before empty C_{60} subtraction.

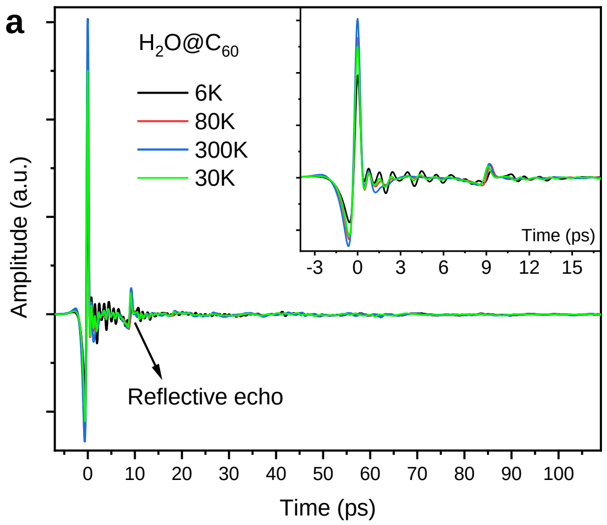

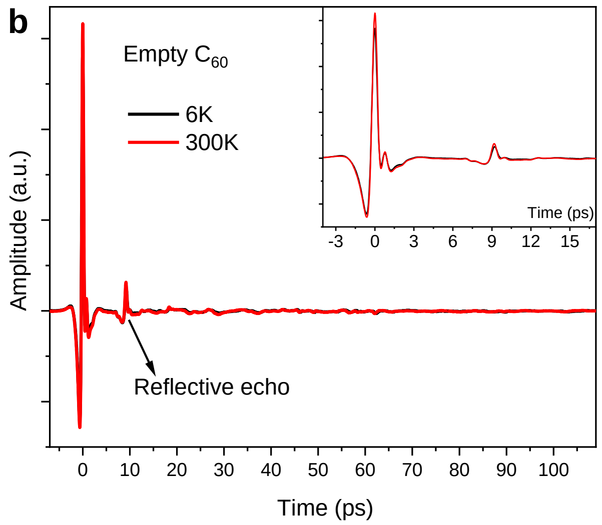

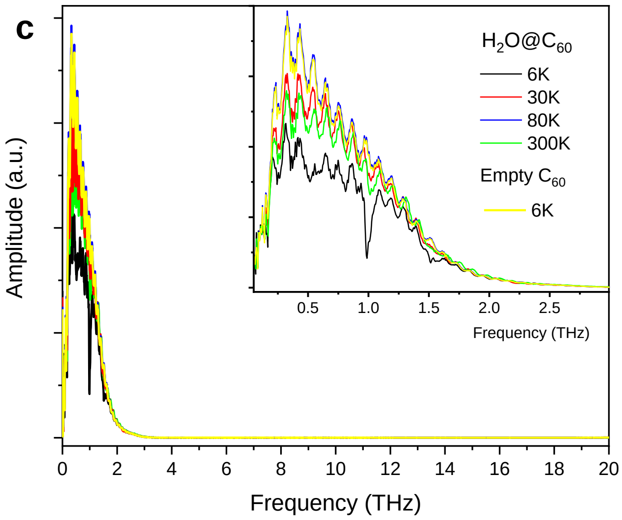

Fig. S1. **a**, Transmitted THz pulses from the $H_2O@C_{60}$ pellet at different temperatures. The inset presents the zooming into the early part of the signals. **b**, Same as panel a, but for the empty C_{60} pellet. The arrows show the internal THz echo signals from the pellet-air interface. **c**, The Fourier spectra of the signals in panel a and b.

Polarization relaxation.

To determine the $\mathbf{P}(t)$ relaxation time constants we fit the measured time domain signals (shown in **Fig. 2a**) by decaying oscillatory functions $\sum_i A_i\, e^{-\gamma t}\sin(2\pi\nu_i t)$. Here, to accurately determine the time constants we use a distribution of frequencies ν_i centered around 0.5 THz, 1 THz, 1.5 THz and 2.2 THz as the initial fit parameters. The left panel of Fig. S2 shows the normalized FIDs at their first peaks at ~1.5 ps and clearly displays different decay behavior of the curves. The polarization time constants as function of temperature extracted from the fitting to the experimental results are shown in the right panel of Fig S2.

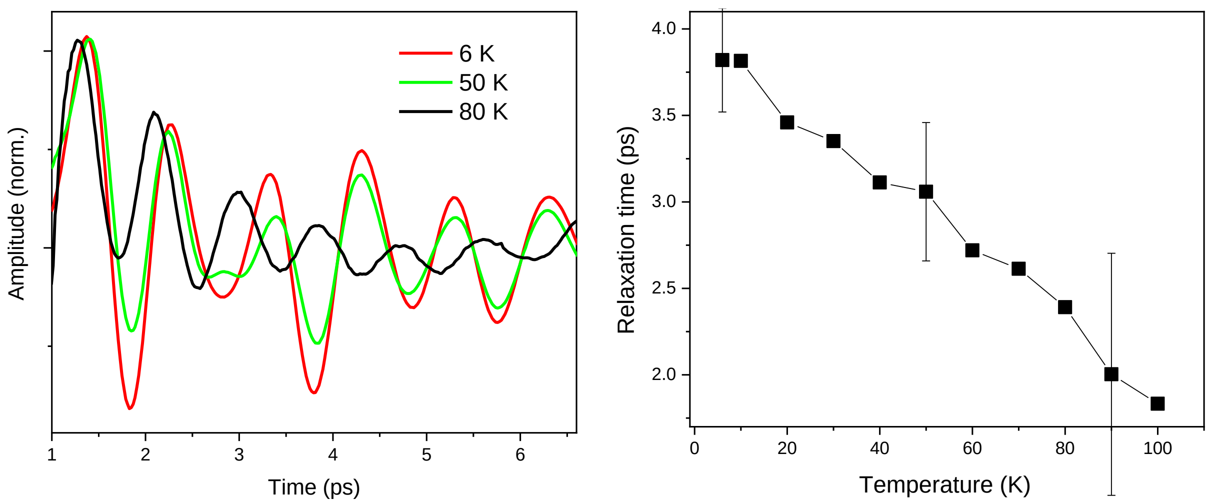

Fig. S2. **Left**, FID emissions of $H_2O@C_{60}$ at temperatures 6 K (red line), 50 K (green line) and 80 K (black line) normalized at their amplitudes at delay time equal to 1.5 ps. **Right**, the decay time constants of FID emissions as function of temperature. The time constants are obtained after fitting the FIDs with decaying sine functions $\sum_i A_i\, e^{-\gamma t}\sin(2\pi\nu_i t)$, where the relaxation times are γ^{-1}.

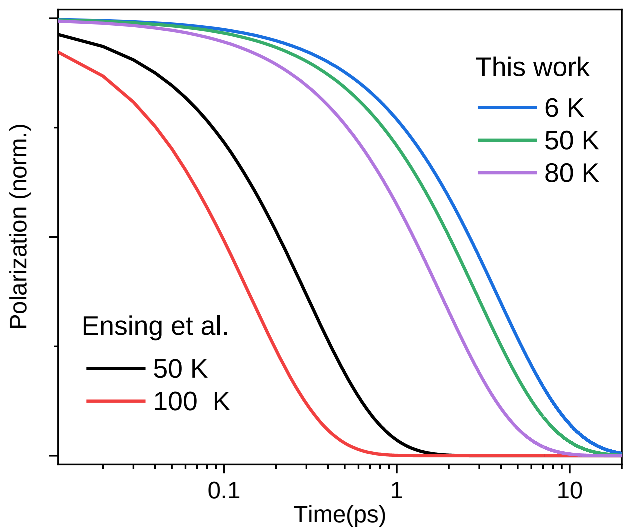

Fig. S3. Comparison between the polarization relaxations obtained from the classical MD simulations (Ensing et al. J. Phys. Chem. A 116, 12184, 2012) at 50 K and 100 K and those obtained from the fit to the experimental FID emissions for temperatures 6 K, 50 K and 80 K. The experimental results clearly show much longer relaxation times and signify the importance of quantum nuclear effects in the dynamics of encapsulated water molecules inside C_{60}.
